# Clinical Evaluation of a Multiplex PCR for the Detection of *Salmonella enterica* Serovars Typhi and Paratyphi A from Blood Specimens in a High-Endemic Setting

**DOI:** 10.4269/ajtmh.18-0992

**Published:** 2019-07-08

**Authors:** Stephane Pouzol, Arif Mohammad Tanmoy, Dilruba Ahmed, Farhana Khanam, W. Abdullah Brooks, Golam Sarower Bhuyan, Laetitia Fabre, Juliet E. Bryant, Marie-Paule Gustin, Philippe Vanhems, Bill Carman, François-Xavier Weill, Firdausi Qadri, Samir Saha, Hubert Endtz

**Affiliations:** 1 Laboratoire des Pathogènes Emergents, Fondation Mérieux, Centre International de Recherche en Infectiologie (CIRI), Lyon, France;; 2 Child Health Research Foundation (CHRF), Dhaka, Bangladesh;; 3 Department of Medical Microbiology and Infectious Diseases, Erasmus MC, Rotterdam, The Netherlands;; 4 International Centre for Diarrhoeal Disease Research (icddr,b), Dhaka, Bangladesh;; 5 Department of International Health, Bloomberg School of Public Health, Johns Hopkins University, Baltimore, Maryland;; 6 Institute for Developing Science and Health Initiatives (ideSHi), Dhaka, Bangladesh;; 7 Unité des Bactéries Pathogènes Entériques, Institut Pasteur, Paris, France;; 8 Department of Public Health, Institute of Pharmacy, University of Lyon 1, Lyon, France;; 9 Service d’Hygiène, Epidémiologie et Prévention, Hôpital Edouard Herriot, Hospices Civils de Lyon, Lyon, France;; 10 Fast Track Diagnostics, Esch sur alzette, Esch-sur-Alzette, Luxembourg

## Abstract

Enteric fever is a major public health concern in endemic areas, particularly in infrastructure-limited countries where *Salmonella* Paratyphi A has emerged in increasing proportion of cases. We aimed to evaluate a method to detect *Salmonella* Typhi (*S.* Typhi) and *Salmonella* Paratyphi A (*S.* Paratyphi A) in febrile patients in Bangladesh. We conducted a prospective study enrolling patients with fever > 38°C admitted to two large urban hospitals and two outpatient clinics located in Dhaka, Bangladesh. We developed and evaluated a method combining short culture with a new molecular assay to simultaneously detect and differentiate *S.* Typhi and *S.* Paratyphi A from other *Salmonella* directly from 2 to 4 mL of whole blood in febrile patients (*n* = 680). A total of 680 cases were enrolled from the four participating sites. An increase in the detection rate (+38.8%) in *S.* Typhi and *S*. Paratyphi A was observed with a multiplex polymerase chain reaction (PCR) assay, and absence of non-typhoidal *Salmonella* detection was reported. All 45 healthy controls were culture and PCR negative, generating an estimated 92.9% of specificity on clinical samples. When clinical performance was assessed in the absence of blood volume prioritization for testing, a latent class model estimates clinical performance ≥ 95% in sensitivity and specificity with likelihood ratio (LR) LR+ > 10 and LR− < 0.1 for the multiplex PCR assay. The alternative method to blood culture we developed may be useful alone or in combination with culture or serological tests for epidemiological studies in high disease burden settings and should be considered as secondary endpoint test for future vaccine trials.

## INTRODUCTION

Enteric fever is a severe systemic infectious disease caused by human-restricted pathogens *Salmonella enterica* serovars Typhi (*S.* Typhi) or Paratyphi (*S.* Paratyphi) A, B, or C. Transmission of the disease is both waterborne and foodborne, or through direct person-to-person contact. The use of antibiotics and improvements in sanitation and hygiene have almost eradicated enteric fever in high-income countries. However, the disease is still of utmost importance in Asia (> 90% cases) and Africa, where nearly 21 million cases and more than 220,000 deaths are estimated to occur annually.^1^ In South Asia, the incidence of enteric fever was 394.2 episodes per 100,000 person-years in 2010,^2^ compared with 240 episodes per 10,000 person-years of enteric fever reported in 2003 in a urban slum of Dhaka, Bangladesh.^3^ Although enteric fever is frequently considered a disease of school children and young adults, population-based studies in Bangladesh have reported the highest incidence rate in children aged less than 5 years (1,870 episodes/100,000 person-years versus 210 episodes/100,000 person-years in older age groups), with higher prevalence of typhoid than paratyphoid.^3,4^

Because of self-medication with antimicrobials before consultations, the classic “textbook” presentation of enteric fever with a slow “step-ladder” rise in fever and toxicity is now rarely seen.^5^ Consequently, differentiating enteric fever in endemic settings from other undifferentiated febrile illnesses, such as influenza, leptospirosis, dengue, or malaria, is very challenging, particularly in children who may present with atypical signs.^6^ The WHO recommends bacterial isolation from blood or bone marrow for definitive diagnosis of enteric fever.^7^ However, because of its invasive nature, bone marrow aspirates are rarely collected, although their culture yields good sensitivity (≈90%) and is relatively refractory to prior antibiotic consumption of patients.^8^ In current clinical practice in most endemic countries, blood culture and Widal test are the most common diagnostic procedures used despite poor sensitivity and specificity.^9^ Indeed, blood culture sensitivity has been estimated at 59% of presumptive cases, ranging from 51% to 65% according to specimen volume,^10^ whereas Widal test is hampered by the lack of standardization of reagents and/or misuse and misinterpretation of results.^11,12^ Serological rapid diagnostic tests such as Typhidot-M or Tubex may represent some improvement over the Widal test, but still have suboptimal sensitivity and specificity.^13^ Another diagnostic assay that shows promise is the TPTest^14^ that detects the *S.* Typhi and *S.* Paratyphi A antibody secreted by isolated lymphocytes.

Polymerase chain reaction (PCR) methods have become central to infectious disease diagnosis as they provide rapid, sensitive, and specific results that are unaffected by patient consumption of antibiotics. In enteric fever diagnosis, molecular tests have been initially developed targeting genes encoding somatic (O), flagellar (H), and Vi antigens of *S.* Typhi,^15–17^ as well as *Salmonella* pathogenicity island 1 (*hilA*),^18^ 16s RNA gene,^19^ or complexes thought to be important for the entry of salmonellae into enterocytes.^20^ In silico comparative genomics and advances in technologies have led to the improvement^21–26^ or development of additional PCR assays.^22,27–30^ Among *S.* Typhi culture–confirmed positives, PCR sensitivity has been generally reported to be > 90% and specificity to be near 100% with a limit of detection (LOD) determined as low as 4 CFU/mL. However, microbiological data suggest that blood culture can be positive in patients with a median bacteremia count of 1 CFU/mL of blood.^31^ *Salmonella* Paratyphi A has emerged in an increasing proportion of enteric fever cases in some settings; the high variability in reported burden estimates in Asia suggests considerable geospatial variability in the burden of paratyphoid fever.^32^ Studies conducted in Dhaka in 2003 indicated that 16.7% cases of enteric fever were related to *S.* Paratyphi A.^3^ Multiplexed PCR assays to simultaneously detect *S.* Typhi and *S.* Paratyphi A are, therefore, needed in Asia; however, the clinical performance of multiplexed PCR assays is frequently compromised relative to monoplex assays in sensitivity (40%)^22,28^ or specificity (63%).^27^

Because PCR performance is highly dependent on the amount of material to be amplified, combining a brief pre-enrichment blood culture with molecular detection is a promising strategy to increase the sensitivity of detection.^33^ In the present study, we developed a rapid assay combining bacterial pre-enrichment by blood culture and multiplex real-time PCR for the detection of *Salmonella* serotypes Typhi and Paratyphi A. We describe the clinical performance of the assay when used to diagnose suspected typhoid patients in Bangladesh.

## MATERIALS AND METHODS

### Design of the molecular assay.

Based on a conventional PCR assay targeting CRISPR regions,^34^ Fast Track Diagnostics (FTD) and Institut Pasteur optimized the length of the amplified regions of *S.* Typhi and *S.* Paratyphi A to suit a multiplex real-time PCR assay format. TaqMan^®^ probes were designed outside the direct repeat region of the CRISPR loci. These were FAM (*S.* Typhi) and Yakima yellow (*S.* Paratyphi A) labeled. *SopD* and *ttrAC* genes were used to define two additional sets of primers/probes (ATTO label) for *S. enterica* (*Salmonella* spp.) identification. Fast Track Diagnostics manufactured the assay under good manufacturing practices and included a positive control (plasmids containing the PCR-targeted regions) and an internal control (inactivated *Streptococcus equi*) for qualification of the extraction procedure (ROX label). Fast Track Diagnostics company commercialized the assay under the FTLYO-35-64-L reference.

### Real-time PCR method.

The samples were expanded to a total volume of 10 mL by adding phosphate-buffered saline (PBS) 1×. Then, 3 μL of an internal control supplied by the manufacturer (FTD, Esch-sur-Alzette, Luxembourg) was added, followed by DNA extraction with QIAamp DNA blood Maxi Kit (Qiagen, Hilden, Germany) according to the manufacturer’s instructions, except that elution was performed with 1 mL of buffer and re-eluted with the first eluate. Subsequently, 10 μL of nucleic acids was subjected to a real-time nucleic amplification using the FTD Enteric Fever Kit (FTD) and the AgPath-ID^™^ One-Step RT-PCR Reagents (Applied Biosystems, Illkirch, France). Polymerase chain reactions were carried out on a CFX96 PCR machine (BioRad, Marnes-la-Coquette, France) using the following conditions: 50°C for 15 minutes, 95°C for 10 minutes followed by 95°C for 8 seconds and 60°C for 34 seconds for 40 cycles. Positive and negative controls provided in the FTD Enteric Fever Kit were performed in the same run. Polymerase chain reaction signals were analyzed using CFX Manager software version 3.1 (BioRad) and expressed as cycle threshold (Ct) values. To validate a result, a serovar-specific signal (either *S.* Typhi or *S.* Paratyphi A) must be associated with the detection of *enterica* species (*Salmonella* spp.), except for non-typhoid *Salmonella* (NTS) isolates which are only positive for the *Salmonella* spp. signal. The comparative PCR test, hereafter designated “Nga-PCR” assay, has been already described by Nga et al.^28^ Briefly, the assay targets *STY0201* and *SSPA2308* genes and was slightly modified as follows: 25-μL reaction volume containing 15 µL of Takyon kit for probe qPCR assays (Eurogentec, Liège, Belgium) with the same primer (0.4 µM) and probe (0.15 µM) set concentration and 10 μL of template DNA. Polymerase chain reactions were carried out on the CFX96 PCR machine (BioRad) using the following conditions: 95°C for 3 minutes followed by 95°C for 10 seconds, 60°C for 30 seconds, and 72°C for 30 seconds for 45 cycles.

### In vitro performance of the molecular assay.

For sensitivity experiments, *S.* Paratyphi A 1K and *S.* Typhimurium LT2 strains from Institut Pasteur (Paris, France) and American Type Culture Collection (Manassas, VA) were, respectively, used. *Salmonella* isolates were grown overnight at 37°C in trypticase soy broth and adjusted to 0.1 OD_600_ unit. After centrifugation (13,000 rpm, 1 minute), bacteria were suspended in 10-fold serial dilutions in PBS and suitable dilutions were then used to seed either whole blood (Etablissement français du sang, Lyon, France) w/wo TSB-ox bile 5% (Beckton Dickinson, Le Pont-de-Claix, France) or PBS. Specificity of detection was evaluated using diverse isolates selected to be representative of global *Salmonella* variation as previously described^34^: 90 *S.* Typhi isolates, 33 *S.* Paratyphi A isolates, and 16 NTS isolates. In addition, 39 different non-*Salmonella* bacterial isolates were tested, representing species frequently involved in non-typhoid enteric disease.

### Study sites and participants.

We selected four study sites in Dhaka, Bangladesh, including two hospitals (Dhaka Shishu Hospital, DSH, and International Centre for Diarrhoeal Disease Research, Bangladesh, icddr,b) and two outpatient clinics located in urban slums of Dhaka (Mirpur and Kamalapur). From August 2014 to June 2015, adults and children aged > 2 years with reported fever > 38°C for three consecutive days were recruited for the study. The exclusion criteria were fever with clinical signs indicating a clear focus of infection, inability to collect the required volume of blood, and refusal to consent. Controls were identified from hospital surgery wards or outpatient wards, with no history of fever post and prior 7 days of blood sampling and informed consent.

Blood culture, identification, and characterization of organisms and pre-enrichment were performed at the laboratories of DSH and icddr,b. External quality assurance program, through the U.K. National External Quality Assessment Service and the American College of Pathologists, is part of the routine quality assurance program. The microbiology laboratory at DSH is a reference laboratory for SEARO-WHO. The clinical microbiology laboratory at icddr,b is accredited against ISO 15189 since 2011.

### Blood collection and culture.

The recommended blood withdrawal volume for testing was stratified into three different groups based on age (2–5, 5–17, and > 17 years). Three milliliters of blood was collected to perform PCR in all age groups, whereas 3 mL (2- to 4-year-old group) to 5 mL was withdrawn for culture. An additional volume (1 mL) was collected to carry out a secondary endpoint test (TPTest, data not included). Moreover, 205 patients consented to participate in an additional study to evaluate an alternative test, and for these individuals, an additional volume of blood (5 mL) was collected.

Blood culture was performed with either the BACTEC (Becton Dickinson and Company) or 3D BacT/Alert (bioMérieux, Marcy-l’Etoile, France) automated culture system at DSH and icddr,b, respectively. The weight of the aerobic blood culture bottle was measured before and after inoculation to determine the precise volume of blood drawn from the patient. The bottle was incubated at 37°C for a period of 5 days as recommended by the manufacturer. At the end of the incubation period, each bottle that was not flagged positive by the blood culture system was inspected visually. Any positive samples were subcultured on sheep blood agar and MacConkey agar plates, and any growth of Gram-negative organisms was further characterized up to the species level by routine biochemical tests and agglutination with Remel agglutinating sera (Thermo Fisher Scientific, Illkirch, France).

### Clinical study laboratory method.

In parallel to blood culture, an additional blood specimen was collected for each participant and mixed with an equivalent volume of TSB-5% bile in a BD Falcon^™^ conical 50-mL tube (Becton Dickinson and Company) at arrival in the microbiology laboratory. The tubes were then incubated for 5 hours at 37°C with shaking before −80°C storage and shipment. DNA extraction and multiplex PCR amplification were performed at the Fondation Mérieux laboratory following the protocol previously described.

### Ethics.

The study was approved by ethical committees of the Bangladesh Institute of Child Health (BICH-ERC-2/6/2012) and icddr,b (PR#13014-27/02/2013).

### Statistical analysis.

Categorical variables were described as number, and continuous variables were described as median and interquartile range (IQR); they were compared, respectively, by the Mann–Whitney U-test or Kruskal–Wallis test in one-way analysis of variance, as appropriate. R (http://www.R-project.org) version 3.4.4 software was used for data computation and analysis. R software with epiR package was used to calculate accuracy and odds ratio of the PCRs with blood culture as gold standard. Bayesian latent class model (LCM) was performed with MPlus software version 7.11 (Muthén & Muthén, Los Angeles, CA).

## RESULTS

### In vitro performance of the multiplex real-time PCR assay.

Using serially diluted *Salmonella* strains spiked in either PBS or whole blood, we assessed the in vitro sensitivity of the multiplex real-time PCR assay (Table 1). All the standard curves demonstrated good analytical performance with *R*^2^ > 0.980 and PCR efficiency in the range of 90–110%. No significant variation in the Ct value was observed on DNA extracted from whole blood or PBS (*P* > 0.2 with nonparametric *t*-test), reflecting no influence of potential blood inhibitors on the PCR assay. The latest dilution with 100% of detected repetitions has been considered to define a reliable LOD. Although able to detect 1 CFU/reaction, the assay demonstrated consistent detection of replicates as low as 10 CFU/reaction for spiked bacteria in PBS and whole blood.

**Table 1:** Detection limit and Ct value comparison of the multiplex real-time polymerase chain reaction (PCR) assays on diluted bacteria-spiked PBS and whole blood

CFU number/reaction												
			10^4^	10^3^	10^2^	10	1	*R*2	Slope	*E*	Intercept	*P*-value
*S.* Typhi (FAM)	PBS	Ct value*	23.6	27.27	30.65	**33.20**	(35.84)	0.992	−3.219	104.5	36.73	0.347
		CV (%)	0.24	0.26	0.93	0.17	(1.08)					
	Blood	Ct value	23.31	26.64	30.07	**32.94**	(35.86)	0.988	−3.234	103.8	38.31	
		CV (%)	0.42	1.88	0.93	1.54	(0.80)					
*S.* Paratyphi A (VIC)	PBS	Ct value*	24.77	28.37	31.53	**34.32**	Neg	0.989	−3.208	105.1	37.79	1
		CV (%)	0.12	0.56	0.18	2.49	/					
	Blood	Ct value*	24.19	27.35	31.4	**34.68**	Neg	0.989	−3.567	90.7	38.31	
		CV (%)	0.41	1.64	1.54	1.13	/					
*S.* Typhimurium (Cy5)	PBS	Ct value*	23.92	27.45	30.71	**33.66**	(36.68)	0.996	−3.248	103.2	37.06	0.272
		CV (%)	0.06	0.26	0.36	1.13	/					
	Blood	Ct value	24.80	28.29	31.49	**34.23**	(35.75)	0.982	−3.149	107.7	37.57	
		CV (%)	2.02	0.94	1.36	1.77	(1.68)					

Ct = cycle threshold; CV = coefficient of variance. R 3.4.4 software was used for calculation of adjusted R-square, slope, and efficacy through a simple linear regression model and for *P*-value determination (nonparametric Wilcoxon’s test).

* Mean Ct value from three individual replicates. Intra-assay variation (CV [%)]) was calculated by measuring the coefficient of variance of the Ct value on at least three individual assays. *R*^2^ = coefficient of determination; Slope = slope of the curve; *E* = amplification efficiency (%); *E* = ([10−^1/slope^] − 1) × 100. Intercept = mean of the Ct value when CFU number/reaction = 0. Data in () are not considered for final analysis in the absence of full detection for replicates.

We also determined whether the molecular assay could detect other *Salmonella* serovars or any non-*Salmonella* bacteria, using clinical isolates and strains frequently identified in blood culture or involved in non-typhoid enteric disease (Supplemental Table 1). Although PCR results demonstrated cross-detection for a few isolates (one *S.* Paratyphi A and two NTS with Ct value > 32), the Ct value for the specific target was low (range 14.4–19.8), indicating a very high bacterial DNA concentration not in line with what could be expected on a biological sample. Consequently, when diluted 10× or 100×, the molecular assay detected 100% of the *S.* Typhi, Paratyphi A, and NTS isolates and no cross-detection with other enterobacteria.

### Evaluation of the multiplex real-time PCR assay in a typhoid-endemic population.

A total of 680 patients with suspected enteric fever and 45 healthy controls were enrolled from four sites in Dhaka, Bangladesh, between 2014 and 2015, including 80.7% (*n* = 549) aged ≤ 17 years (Table 2). All 45 healthy controls remained negative with both PCR and culture. Results based on blood culture indicated a 14% (98/680) prevalence of the disease. Polymerase chain reaction positivity was significantly correlated with shorter fever duration at the time of presentation (*P* < 0.01) but not with age, nor with differences in the sample volume used for culture or molecular detection. Briefly, 136/680 suspected cases (20%) were positive by PCR (118 *S.* Typhi and 18 *S.* Paratyphi A) with no discrepancy in identification, whereas 98 (14%) were blood culture positive (79 *S.* Typhi and 19 *S.* Paratyphi A). Seven culture-positive samples (four *S.* Typhi and three *S.* Paratyphi A) were not identified with PCR (1%) and exhibited a longer time to positivity in culture (*P* < 0.005). The assay showed the capacity to detect additional samples (43 *S.* Typhi and two *S.* Paratyphi A) in negative blood culture samples.

**Table 2:** Distribution of clinical and laboratory characteristics of patients with fever according to the polymerase chain reaction (PCR) assay results

Characteristics	Suspected cases			Controls	*P*-value (suspected vs. controls)
	PCR+ (*n* = 136)	PCR− (*n* = 544)	*P*-value	*n* = 45	
Categorical variables, *N* (%)					
BC positive	91 (13.4)	7 (1.0)	–	0	–
BC negative	45 (6.6)	537 (79.0)	–	45	–
Continuous variables, median [IQR]					
Age (years)	7.5 [4.5–11]	7.5 [4.6–14]	0.56*	8 [5–10]	0.90*
Temperature (°C)	39.0 [38.3–39.4]	38.8 [38.1–39.4]	0.89	N/A	N/A
Time from fever onset to sample collection (days)	5 [4–7]	7 [4–11]	< 0.001*	N/A	N/A
Blood input for culture (mL)	2.5 [2.0–3.4]	2.6 [2.0–3.4]	0.92	2.3 [1.8–3.3]	0.19
Blood input for PCR (mL)	3.4 [3.2–3.7]	3.4 [3.2–3.7]	0.47	3.4 [3.2–3.6]	0.47
Time to positive BC (hours)	17.0 [14.5–23.0]	32.4 [24.6–36.3]	< 0.001*	N/A	N/A
Volume BC–volume PCR (mL)	−0.9 [−1.6 to +0.2]	−0.9 [−1.6 to 0.0]	0.81	−1.04 [−1.77 to 0.00]	0.55

BC = blood culture.

* Fisher's exact test of independence. Significantly different (*P* < 0.05) categorical variables were described as number and percentage, and continuous variables as median and interquartile range [IQR]. They were compared using the Student *t*-test or Wilcoxon test two-way analysis of variance, as appropriate.

Before evaluating performance in the clinical setting, we benchmarked the multiplex PCR assay with the only similar test available in 2014^28^ (Table 3), hereafter designated the “Nga-PCR” assay. Nga-PCR detected additional (*n* = 49) cases in negative blood culture samples, but, similarly to the multiplex PCR assay, Nga-PCR also failed to detect the same seven culture-positive samples that required longer incubation times, mentioned previously. The Nga-PCR assay identified 81/98 multiple PCR–confirmed cases, especially notable for *S.* Typhi (65/79 versus 75/79), but failed in typing four samples because of a specific signal identification for both *S.* Typhi and *S.* Paratyphi A. Assuming that the blood culture criterion was the best reference standard, we finally determined clinical performance of PCR assays. Both tests showed similar specificity (> 90%) and posttest probabilities associated with large or moderate usefulness of likelihood ratios (LR+ > 9.8 and LR− < 0.2) but a significant difference in specificity (92.9% versus 82.6% for multiplex PCR and Nga-PCR, respectively).

**Table 3:** Multiplex polymerase chain reaction (PCR) assay performance in a typhoid-endemic population in a clinical setting

	Suspected enteric fever cases (*n* = 680) | comparator (true positive) = blood culture										
	TP	FP	FN	TN	Se (%)	Spe (%)	LR+	LR−	DOR	PT+ (%)	PT− (%)
Multiplex PCR assay	91	45	7	537	92.9	92.3	12.01	0.08	155.8	66	1
Nga multiplex assay	81‡	49	17*	533†	82.6	91.6	9.82	0.19	51.8	62	3

DOR = diagnostic odds ratio; FN = false negative; FP = false positive; Se = sensitivity; Spe = specificity; LR+ = positive likelihood ratio; LR− = negative likelihood ratio; PT+ = positive posttest probability; PT− = negative posttest probability; TN = true negative; TP = true positive. Performances in the clinical setting (CI = 95%) were calculated with R3.4.4 software and epiR package.

* Including one undetermined sample (both specific positive signal with *S.* Typhi and *S.* Paratyphi A).

† Including three undetermined samples.

‡ Including one discrepant result with BC.

### Impact of differences in blood volumes used for testing.

**Figure 1.**
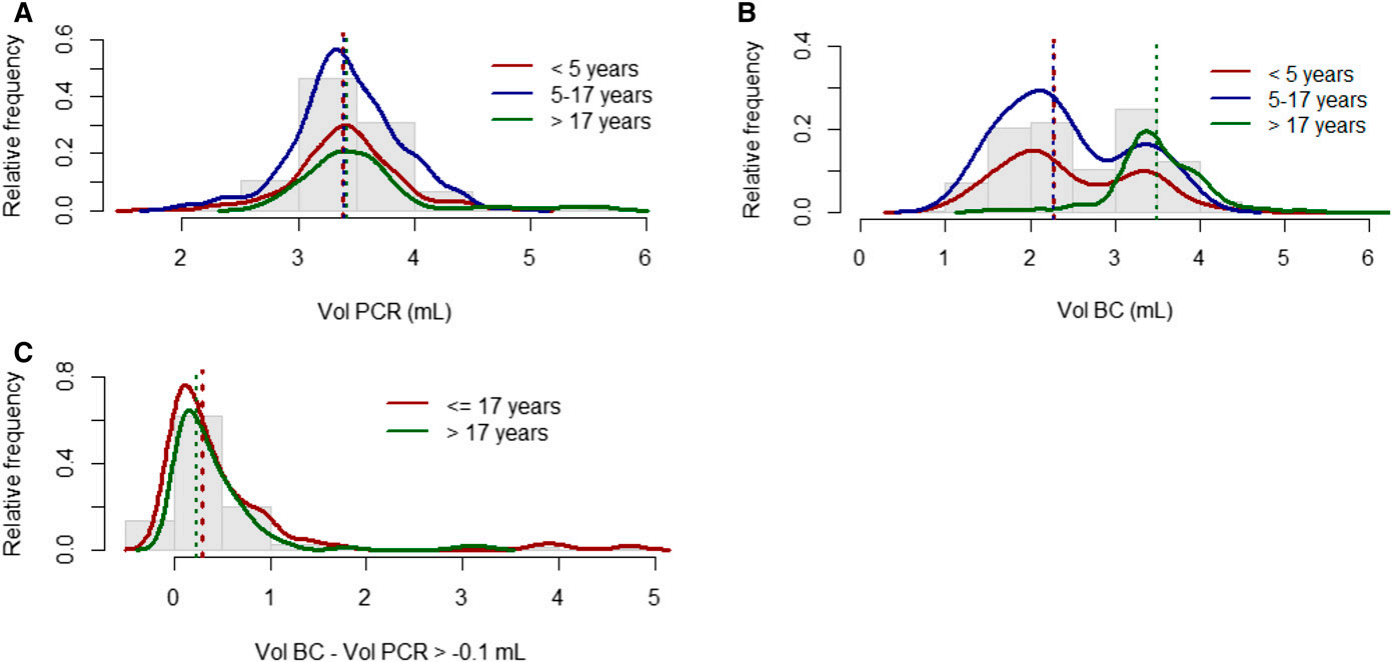
Analysis of the sample volumes used for culture and polymerase chain reaction (PCR) clustered by patient age. Histograms (gray bars) show relative frequency in volumes, and lines are the related tendency curve for the respective age groups. A and B represent distribution of collected volumes used for either PCR or culture. Red, blue, and green lines indicate the population of patients aged ≤ 5 years, 5–17 years, and older, respectively. The corresponding colored dash lines indicate the median volume used for testing. Whereas they all appear superimposed on A, only under 5 and 5–17 years old age groups are similar in B. C shows the distribution of samples with a volume difference between culture and PCR ≥ -0.1 mL. Red line is the related tendency curve for the ≤ 17 years age group (green for adults), and the corresponding colored dash lines indicate the median difference in volume by cluster of age. BC = blood culture; Vol = volume.

The average blood volume used for PCR (3.4 mL) was similar among all age groups (Figure 1A). In 11.6% of the total number of patients, a blood volume ≤ 3.0 mL was drawn. However, the average volume of blood used for culture was larger in adults than in children aged ≤ 17 years (3.5 mL versus 2.3 mL, respectively) (Figure 1B). In 29.9% (164/549) of children aged ≤ 17 years, a blood culture was performed with a volume > 3.0 mL. As the average blood volumes used for PCR and culture in patients aged ≤ 17 years were similar, we merged the cohorts in the further analysis. However, to control for potential bias due to different volumes used for PCR versus culture, we also separately analyzed all patients for which the difference in volume between PCR and culture was greater than −0.1 mL (Figure 1C and Table 4). A total of 214 cases (54% children, 46% adults) have been selected with higher average volume for blood culture (3.6 mL) than PCR (3.3 mL). This subset included six of seven culture-positive specimens that were PCR negative. In nine children aged ≤ 17 years, the blood volumes drawn for culture and PCR were less than 3 mL. For this subset, the performance of the multiplex PCR demonstrated a drop in sensitivity (92.9–82.4%) and specificity (92.3–90.0%), with better performances observed among children aged ≤ 17 years than adults (Table 4).

**Table 4:** Multiplex PCR assay performance in samples with a difference in volume used for culture and PCR ≥ −0.1 mL

	Volume BC–volume PCR ≥ −0.1mL | comparator (true positive) = blood culture										
Multiplex PCR assay	TP	FP	FN	TN	Se (%)	Spe (%)	LR+	LR−	DOR	PT+ (%)	PT− (%)
All ages (*n* = 214)	28	18	6	162	82.4	90.0	8.24	0.20	42.0	61	4
Age ≤ 17 years (*n* = 123)	20	9	3	91	87.0	91.0	9.66	0.14	67.4	69	3
Age > 17 years (*n* = 91)	8	9	3	71	72.7	88.8	6.46	0.31	21.0	48	4

DOR = diagnostic odds ratio; FP = false positive; FN = false negative; LR+ = positive likelihood ratio; LR− = negative likelihood ratio; PCR = polymerase chain reaction; PT+ = positive posttest probability; PT− = negative posttest probability; Se = sensitivity; Spe = specificity; TP = true positive; TN = true negative. Performances in the clinical setting (CI = 95%) were calculated with R3.4.4 software and epiR package.

Clinical performance estimated by LCM indicated 73% sensitivity (including 69% in adults) and 99% specificity in all age groups for culture (Table 5). Both PCR assays indicated > 90% sensitivity and specificity as well as LR+ > 10 and LR− < 0.1 in adults. When assessed in the absence of difference in blood volume used for testing (Table 6), LCM estimates reported a decrease in culture sensitivity (73–69%) as a result of a drop in older patients (61%). By contrast, performance for both PCR assays indicated a rise in sensitivity in all age groups, except in adults with Nga-PCR (93–90%). No or few variations in specificity for PCR assays were observed.

**Table 5:** Results of latent class model estimates (95% CI)

	Overall population (n = 680)			
All ages	Se	Spe	LR+	LR−
Culture	0.73	0.99	64.6	0.27
Multiplex PCR assay	0.99	0.98	45.7	0.01
Nga-PCR assay	0.89	0.96	22.02	0.11
Age ≤ 17 years	Se	Spe	LR+	LR−
Culture	0.74	0.99	100	0.26
Multiplex PCR assay	0.94	0.98	45.5	0.01
Nga-PCR assay	0.88	0.97	34.5	0.12
Age > 17 years	Se	Spe	LR+	LR−
Culture	0.69	0.98	28.6	0.31
Multiplex PCR assay	1.0	0.98	43.5	∞
Nga-PCR assay	0.93	0.91	10.5	0.07

PCR = polymerase chain reaction; LR+ = positive likelihood ratio; LR− = negative likelihood; Se = sensitivity; Spe = specificity. Estimates (CI= 95%) were calculated with MPlus software version 7.11. Without PCR prioritization means difference in volume (volume BC–volume PCR) ≥ −0.1mL. For computational reason, we selected only samples between 3 mL and 4 mL of blood for culture, eliminating, therefore, 36 samples.

**Table 6:** Results of latent class model estimates (95% CI)

	Overall population without PCR prioritization (*n* = 214)			
All ages	Se	Spe	LR+	LR−
Culture	0.69	0.96	19.2	0.32
Multiplex PCR assay	1.0	0.97	33.3	∞
Nga-PCR assay	0.9	0.95	17.0	0.10
Age ≤ 17 years	Se	Spe	LR+	LR−
Culture	0.75	0.99	109.1	0.25
Multiplex PCR assay	0.99	0.98	46.4	0.01
Nga-PCR assay	0.89	0.97	32.6	0.11
Age > 17 years	Se	Spe	LR+	LR−
Culture	0.61	0.97	22.7	0.40
Multiplex PCR assay	1.0	0.98	41.7	∞
Nga-PCR assay	0.90	0.91	10.1	0.10

LR+ = positive likelihood ratio; LR− = negative likelihood ratio; PCR = polymerase chain reaction; Se = sensitivity; Spe = specificity. Estimates (CI = 95%) were calculated with MPlus software version 7.11. Without PCR prioritization means difference in volume (volume BC − volume PCR) ≥ −0.1mL. For computational reason, we selected only samples between 3 mL and 4 mL of blood for culture, eliminating, therefore, 36 samples.

## DISCUSSION

The assay described here was designed for the simultaneous identification and differentiation of *S.* Typhi, *S.* Paratyphi A, and NTS. The molecular basis of the present assay was identified through in silico analysis of *S. enterica* CRISPR loci polymorphism^34^ and consists of unique and constant spacers in *Salmonella* serovars Typhi and Paratyphi A that are suitable for distinguishing the two serovars. All the standard curves generated for analytical performance indicated an *R*^2^ > 0.980 and a PCR efficiency in the range of 80–120%, therefore meeting the requirements to validate a qualitative multiplex qPCR assay.^35^ The assay demonstrated robust specificity similar to other studies,^22,28^ with no cross-detection in isolates and absence of detection in controls. The LOD was 10 copies/reaction, equivalent to 1 copy/μL when compared with the final elution volume.

Additional case identification with PCR methods has been previously reported from large cohorts with low (< 15%), moderate, or high (≥ 85%) sensitivity rates.^8^ In this study, detection rates were low in culture negatives, as low as 7.7% (45/582) and 8.5% (49/582) with the multiplex PCR and Nga-PCR assays, respectively. The addition of bile in enrichment media to release bacteria from the blood intracellular compartment has been shown to produce an almost 2-fold rise in bacterial numbers^36^ that may explain the rise in PCR identification. An alternative explanation includes the use of lower than recommended volumes in culture that may adversely affect recovery and/or detection times, therefore highlighting suboptimal culture sensitivity. However, in the absence of a secondary endpoint test, we do not have an independent confirmation of infection or past exposure in the PCR-positive detections among the culture-negative samples.

Despite good overall sensitivity of the PCR assay, we and others^27,28,37^ failed to detect *S.* Typhi/Paratyphi A in seven of the 98 culture-positive samples. Assay failure for those cases could not be attributed to sample volume used for testing nor to the genetic diversity of the bacterial strains because the cultured strains were subsequently identified by PCR (data not shown). The longer time to positivity in these samples suggests very low initial bacteremia and/or the presence of antibiotics. Some commercial blood culture bottles may contain resins and other antibiotic neutralizing substances (beads), thereby increasing the pathogen recovery in patients on antibiotic therapy compared with non-resin–based culture media.^38^ In the absence of antibiotic-binding substances in the pre-enrichment media, the growth of bacteria during the 5-hour incubation period might be inhibited, resulting in a bacterial inoculum below the detection limit of PCR. Moreover, considering the low median reported concentration of *S.* Typhi in blood^39^ (0.3–1 CFU/mL) and the potential low bacteremia in the specimen, discrepant samples may be explained by the absence of bacteria in aliquots.

An important limitation of this study was the generally low blood volume used for culture (average 2.5 mL) compared with routine practice, especially but not restricted to adult participants. The use of a limited volume of blood (2–3 mL) for cultures from children is a current practice despite better bacterial growth with large (> 5 mL) volume of blood.^31^ In addition, although high and repeated volumes are associated with higher probability of positivity in culture,^9^ none of the 13 positive cultures in adults have been performed with more than 4 mL of blood. However, despite assumed suboptimal conditions, culture results relative to PCR indicated 67% sensitivity in the overall population (Supplemental Table 2), in line with data reported in a recent review.^40^ The issue of blood volume and culture sensitivity underscore the need for newer, more sensitive assays for *Salmonella* that can be performed at these lower volumes, highlighting the rationale for studies such as ours. Thus, the proportion of positive blood cultures was not significantly different (chi-squared test) depending on whether culture was performed on < 2 mL (28/187), 2–3 mL (31/217), or 3–4 mL (38/253) of blood, nor related to age (85/542 in children aged < 17 years and 13/138 in adults).

Because sites with high typhoid endemicity are the same ones where obtaining blood specimens at higher volumes is a challenge, blood culture remains a poor performer under real-world conditions and could not be considered as a satisfactory gold standard. Latent class analysis is a model-based approach to approximate the sensitivities and specificities of different tests in the absence of a reliable gold standard.^41^ Thus, LCM considers that each test could be imperfect in diagnosing the true disease status and estimates which of these assays are the best performers under conditions and limitations of this study. The true disease status of the patient population is then defined on the basis of overall prevalence (the probability that a patient with suspected typhoid fever is truly infected with *S.* Typhi). The latent class model indicates 73% sensitivity in the overall population (74% in children aged less than 17 years), in the high range of reported estimates.^10^ However, in the absence of a secondary endpoint test to confirm PCR results, there is a non-neglectable risk of class membership misclassification (confirmed/suspected) in addition to the unbalanced weight of the respective tests. This might be counterbalanced by running the model with results from bone marrow culture and/or serological test results.

Despite short turnaround times, additional sensitivity benefit, and capacity to serotype, the utility of PCR-based tests for enteric disease detection is still under debate. This study provided clinical performance results for a new assay that combined culture incubation steps with multiplex real-time PCR. Further evaluations are needed to compare the performance of the assay relative to bone marrow culture and to repeat clinical evaluation in other geographical areas where NTS is more prevalent. Our multiplex PCR assay demonstrated robust improvements in detection over blood culture, and performance was best in children for which blood sample volumes were limited. Additional developments have made it possible to market a ready-to-use PCR kit (freeze-dried) that uses a larger volume of input DNA extract (15 μL). As molecular diagnostics are becoming increasingly accessible in developing country clinical laboratories, this new tool may be useful alone or in combination with other assays, particularly in the context of high disease burden, and for use in epidemiological studies and as a secondary endpoint test for future vaccine trials.

## Supplemental materials

Supplemental tables
