# Supplementary material for: Clinical Evaluation of a Multiplex PCR for the Detection of Salmonella enterica Serovars Typhi and Paratyphi A from Blood Specimens in a High-Endemic Setting
Source: Am J Trop Med Hyg. 2019 Jul 8;101(3):513–20. doi: 10.4269/ajtmh.18-0992 (PMC6726943; doi:10.4269/ajtmh.18-0992)
Supplement: Supplementary file 1 [file tpmd180992.SD1.pdf]

Supplementary Table 1: Specificity results of multiplex real-time PCR assay

**S. enterica subsp. enterica serovar (Source)**	No. of Strains Tested	*S.* Typhi multiplex qPCR	*S.* Paratyphi A multiplex qPCR	*S.* spp multiplex qPCR
Multiple *Salmonella* Typhi strains and clinical isolates (FNRC-ESS; WHO-SALM)	90	+	-	+
Multiple *Salmonella* Paratyphi A strains and clinical isolates (FNRC-ESS; WHO-SALM)	33	- (+)	+	+
Blegdam (WHO-SALM)	1	-	-	+
Blockley (WHO-SALM)	1	-	-	+
Canada (WHO-SALM)	1	-	-	+
Choleraesuis var. Decatur (WHO-SALM)	1	-	-	+
Dublin (WHO-SALM)*	1	- (+)	-	+
Emek (WHO-SALM)	1	-	-	+
Enteritidis (WHO-SALM)*	1	- (+)	-	+
Indiana (WHO-SALM)	1	-	-	+
Manhattan (WHO-SALM)	1	-	-	+
Mbandaka (WHO-SALM)	1	-	-	+
Mikawasima (WHO-SALM)	1	-	-	+
Muenchen (WHO-SALM)	1	-	-	+
Nitra (WHO-SALM)	1	-	-	+
Paratyphi B (WHO-SALM)	2	-	-	+
Paratyphi C (WHO-SALM)	1	-	-	+
Typhimurium (WHO-SALM)	1	-	-	+
**Others species**				
Enterobacter cloacae sbsp. *Cloacae* (CIP)	1	-	-	-
*Escherichia coli (*FNRC-ESS)*	3	-	-	- (+)
Escherichia vulneris (CIP)	1	-	-	-
Neisseria meningitidis (FNRC-Neisseria)	1	-	-	-
Serratia marcescens (CIP)	1	-	-	-
*Shigella dysenteriae (*FNRC-ESS; CIP)	2	-	-	-
Shigella flexneri 2a (FNRC-ESS)	1	-	-	-
Staphylococcus aureus (CIP)	1	-	-	-
Staphylococcus epidermidis (CIP)	1	-	-	-
Streptococcus agalactiae (CIP)	1	-	-	-
Streptococcus pneumoniae (CIP)	1	-	-	-
Streptococcus pyogenes (CIP)	1	-	-	-
Budvicia aquatica (CIP)	1	-	-	-
Citrobacter freundii (CIP)	1	-	-	-
Citrobacter gillenii (CIP)	1	-	-	-
Citrobacter koseri (CIP)	1	-	-	-
Citrobacter youngae (CIP)	1	-	-	-
Enterobacter cloacae sbsp. *dissolvens* (CIP)	1	-	-	-
Enterobacter sakasaki (CIP)*	1	-	-	- (+)
Escherichia albertii (CIP)	1	-	-	-
Escherichia hermanii (CIP)	1	-	-	-
Ewingella americana (CIP)	1	-	-	-
Hafnia alvei (CIP)	1	-	-	-
Klebsiella oxytoca (CIP)	1	-	-	-

Klebsiella pneumoniae sbsp. *Pneumoniae* (CIP)	1	-	-	-
Klebsiella pneumoniae sbsp. *rhinoscleromatis* (CIP)	1	-	-	-
Leclercia adercarboxycola (CIP)	1	-	-	-
Proteus mirabilis (CIP)	1	-	-	-
Plesiomonas shigelloides (CIP)	1	-	-	-
Pectobacterium atrosepticum (CIP)	1	-	-	-
Raoultella planticola (CIP)	1	-	-	-
Rahnella aquatilis (CIP)	1	-	-	-
Serratia fonticola (CIP)	1	-	-	-
Xenorhabdus japonica (CIP)*	1	-	-	- (+)
Yersinia enterocolitica sbsp. *enterocolitica* (CIP)	1	-	-	-
Yersinia pseudotuberculosis sbsp. *Pseudotuberculosis (*CIP)	1	-	-	-

FNRC-ESS, French National Reference Center for *Escherichia coli-Shigella-Salmonella*, Institut Pasteur; WHO-Salm, World Health Organization collaborative center for reference and research on *Salmonella*, Institut Pasteur*;* CIP= Collection de l'Institut Pasteur; FNRC-Neisseria=French National Reference Center for *Neisseria.* (*) = cross-detection observed for some strains at high DNA concentration (negative when 10X/100X diluted) with Ct Value > 33.5.
