# Supplementary material for: Clinical Evaluation of a Multiplex PCR for the Detection of Salmonella enterica Serovars Typhi and Paratyphi A from Blood Specimens in a High-Endemic Setting
Source: Am J Trop Med Hyg. 2019 Jul 8;101(3):513–20. doi: 10.4269/ajtmh.18-0992 (PMC6726943; doi:10.4269/ajtmh.18-0992)
Supplement: Supplementary file 2 [file tpmd180992.SD2.pdf]

Supplementary Table 2: Determination of Blood Culture performance.

A) **Overall population | Comparator (True positive) = Multiplex PCR Assay**

Blood Culture	**TP**	**FP**	**FN**	**TN**	**Se (%)**	**Spe (%)**	**LR+**	**LR-**	**DOR**	**PT+ (%)**	**PT- (%)**
All (n=680)	91	7	45	537	66.9	98.7	52.0	0.34	155.1	93	8
≤ 17 years (n=549)	81	4	36	428	69.2	99.1	74.8	0.31	240.8	95	8
> 17 years (n=131)	10	3	9	109	52.6	97.3	19.6	0.49	40.4	77	8

B) **Volume BC – Volume PCR ≥ -0.1mL | Comparator (True positive) = Multiplex PCR Assay**

Blood Culture	**TP**	**FP**	**FN**	**TN**	**Se (%)**	**Spe (%)**	**LR+**	**LR-**	**DOR**	**PT+ (%)**	**PT- (%)**
All (n=214)	28	6	18	162	60.9	96.4	17.0	0.41	42.0	82	10
≤ 17 years (n=123)	20	3	9	91	69.0	96.8	21.6	0.32	67.4	87	9
> 17 years (n=91)	8	3	9	71	47.1	95.9	11.6	0.55	21.0	73	11

Legend: TP, True Positive; FP, False Positive; TN, True Negative; FN, False Negative; Se, sensitivity; Spe, specificity; LR+, Positive Likelihood Ratio; LR-, Negative Likelihood Ratio; DOR, Diagnostic Odds Ratio; PT+, Positive Post-test probability; PT-, Negative Post-test probability.

Supplementary Table 2 shows analysis of blood culture performance in suspected cases with (A)/without (B) difference in volume (Volume BC – Volume PCR) ≥ -0.1mL. Performances in clinical setting (confidence interval = 95%) were calculated with R3.4.4 software and epiR package.
